# Choroidal morphology and microvascular structure in eyes of patients with idiopathic normal pressure hydrocephalus before and after ventriculo-peritoneal shunt surgery

**DOI:** 10.1038/s41598-023-43518-8

**Published:** 2023-09-29

**Authors:** Nicola Valsecchi, Matilde Roda, Simone Febbraro, Diana Wrona, Giulia Marega, Giorgio Palandri, Giulia Giannini, David Milletti, Costantino Schiavi, Luigi Fontana

**Affiliations:** 1 Ophthalmology Unit, Dipartimento di Scienze Mediche e Chirurgiche, Alma Mater Studiorum University of Bologna, Bologna, Italy; 2 IRCCS Azienda Ospedaliero-Universitaria di Bologna, Via Pelagio Palagi 9, 40138 Bologna, Italy; 3 Unit of Neurosurgery, IRCCS Istituto delle Scienze Neurologiche di Bologna, Bologna, Italy; 4 Unit of Neurology, IRCCS Istituto delle Scienze Neurologiche di Bologna, Bologna, Italy; 5 Department of Biomedical and Neuromotor Sciences (DIBINEM), University of Bologna, Bologna, Italy; 6 Unit of Rehabilitation Medicine, IRCCS Istituto delle Scienze Neurologiche di Bologna, Bologna, Italy

**Keywords:** Hydrocephalus, Diagnostic markers, Retina

## Abstract

The present study aims to investigate the choroidal morphology and microvascular structure in eyes of patients with idiopathic normal pressure hydrocephalus (iNPH) compared with the eyes of healthy age-matched individuals, and to assess the choroidal structure in eyes of iNPH patients before and after shunt surgery using Optical Coherence Tomography (OCT). The primary objective was to assess the choroidal morphology in eyes of iNPH patients before and after ventriculo-peritoneal (VP) surgery compared to age and sex-matched healthy individuals. The secondary objective was to compare the choroidal morphology of iNPH patients before and after a mean of 56 days from shunt surgery. Eighteen consecutive patients diagnosed with iNPH and 18 healthy controls were prospectively recruited between November 2021 and October 2022. Spectral-domain optical coherence tomography (SD-OCT) with enhanced depth imaging (EDI) was conducted before and within 4 months after shunt surgery. Images were binarized using the ImageJ software, and the choroidal vascular index (CVI) was calculated. Sub-foveal choroidal thickness (SFCT), total choroidal area (TCA), luminal choroidal area (LCA), and stromal choroidal area (SCA) were significantly increased in iNPH patients before surgery compared to the control group (*p* < 0.05). SFCT, TCA, and SCA were significantly increased in iNPH patients after surgery compared to the control group (*p* < 0.05). There were no differences in the CVI between iNPH patients and controls. No statistical differences in the choroidal structure were observed before and after VP shunt surgery (*p* > 0.05). In conclusion, the choroid was thicker in iNPH patients before and after VP shunt compared to age-matched healthy individuals. However, there were no difference in the choroidal microstructure in the eyes of iNPH patients before and after a mean of 3 months from VP shunt surgery.

## Introduction

Idiopathic normal pressure hydrocephalus (iNPH) is a neurological condition clinically characterized by the progressive onset of gait disturbances, cognitive impairment, and urinary incontinence^1^. The hallmarks of the syndrome are the presence of ventricular dilation at neuroimaging combined with normal cerebral spinal fluid pressure.

iNPH is a disease of the elderly population, with a prevalence of 0.2% in patients aged 70–79 years and 5.9% in those older than 80 years^2^. The treatment of choice in iNPH patients consists of cerebrospinal fluid (CSF) shunting, to alleviate symptoms caused by dilated ventricles, and ventriculoperitoneal (VP) shunt is the widest surgical procedure performed in patients with iNPH, with a success rate of up to 85% after 12 months from shunt surgery^3–5^.

To date, the exact pathogenesis of iNPH is still controversial. Reduction in CSF drainage, neuroinflammation, vascular hypoperfusion, and impaired glymphatic circulation are thought to play a role in the development of iNPH^6,7^.

A recent study reported choroidal alterations in eyes of NPH patients using Spectral domain-optical coherence tomography (SD-OCT) imaging, supporting the hypothesis of choroidal susceptibility to hemodynamic changes in iNPH^8^. The choroid is the most vascular tissue in the eye. It lies between the retina and the sclera, and its main function is to nourish the retinal photoreceptors and to supply some regions of the optic nerve head. It receives approximately 95% of the ophthalmic artery blood, through the short posterior ciliary arteries, and it can change its volume dramatically according to the requirements of the eye metabolism^9^. The drainage of the venous blood is carried out through the vortex veins, and then into the intracranial cavernous sinus. Thus, alterations of the venous drainage in a context of impaired CSF dynamic might determine consequences in the choroidal vasculature. Moreover, the choroid is composed of neural tissues, melanocytes, fibroblasts, macrophages, dendritic cells, and vessels, implicated in many inflammatory processes. SD-OCT is a non-invasive diagnostic tool that enables the assessment of the retina and the choroidal morphology. Moreover, the enhanced depth imaging (EDI) function allows the assessment of the choroidal vascular index (CVI), a new quantitative parameter for measuring the microvascular structure of the choroid^10^. Therefore, the goal of the present study was to investigate the microvascular structure of the choroid in eyes of iNPH patients, and to understand if shunt surgery may determine alterations in the choroidal morphology.

## Material and methods

### Study population

Twenty iNPH patients were recruited between November 2021 and October 2022 from an ongoing prospective study at the Unit of Ophthalmology, University of Bologna, in collaboration with the Bologna PRO-HYDRO study group of the Istituto delle Scienze Neurologiche di Bologna. The study was adherent to the tenets of the Declaration of Helsinki and was approved by the Institutional Review Board/Ethics Committee of the local health service of Bologna, Italy (Cod CE: 809/2021). Written informed consent was obtained from all subjects included in the study.

The multidisciplinary Bologna PRO-HYDRO study group discussed each patient’s case and indications for surgery. All patients included in this study fulfilled the criteria for diagnosis of iNPH and received an indication for VP shunt surgery^11,12^.

We imaged the choroidal morphology of the eye with the best OCT image resolution in iNPH patients before and after the implant of CODMAN HAKIM programmable valves with the SIPHONGUARD system (Integra LifeSciences, Manfield, Massachusetts, USA), and we compared the choroidal structure with a cohort of 18 healthy, age and matched healthy controls.

The primary objective of the present study was to assess the choroidal morphology using SD-OCT in eyes of iNPH patients before and after VP shunt surgery compared to age and sex-matched healthy individuals.

The secondary objective of the study was to compare the choroidal morphology of iNPH patients before and within 4 months from shunt surgery, to evaluate the role of VP shunt surgery in the variation of choroidal microstructure in the early postoperative time.

We excluded iNPH patients with an ophthalmic history of ocular trauma, retinal detachment, corneal opacities, advanced cataract, age related macular degeneration, diabetic retinopathy, high myopia (> − 6 diopters), axial length (AL) > 26 mm and < 21 mm, and history of uveitis. Glaucoma was not considered an exclusion criterion, as an association between iNPH and Open-Angle Glaucoma has been previously described^13^.

The control group was formed by healthy subjects of age comprised between 65 and 85 years, and were selected according to the same exclusion criteria described for the iNPH group.

### Clinical assessment

Each iNPH patient underwent a comprehensive eye examination including best-corrected visual acuity (BCVA) assessment reported in logarithm of the minimum angle of resolution (LogMar), slit lamp examination of the anterior segment, indirect ophthalmoscopy, Goldmann applanation tonometer, assessment of axial length with Lenstar (Haag-Streit, Koniz, Swiss), and OCT Macula.

All OCT examinations were carried out using the same Heidelberg Spectralis SD-OCT (Heidelberg Engineering, Heidelberg, Germany) apparatus. The information regarding medical therapies and comorbidities was collected during the baseline ophthalmic evaluation.

### OCT image acquisition

Each acquisition was performed between 11.00 and 14.00 to avoid diurnal variations. Macular OCT images were acquired with enhanced depth imaging (EDI) mode using a volume scan of 30 × 10° centered on the fovea.

**Figure 1:**
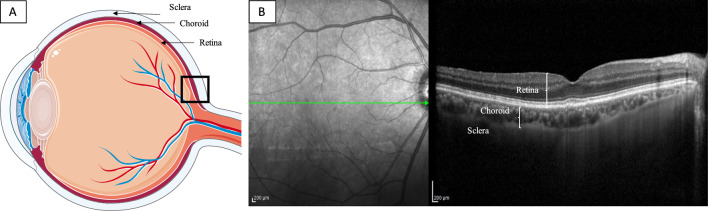
Eye anatomy and OCT acquisition. (**A**) A schematic representation of the eye anatomy is shown. The choroid is the vascular layer that lies between the retina and the sclera. Its main function is to nourish retinal photoreceptors. (**B**) The OCT scan centered on the fovea is shown.

The OCT scan passing through the central foveal region was chosen for the analysis. A schematic diagram of the eye anatomy and an OCT acquisition is shown in Fig. 1. See Fig. 1.

The sub-foveal choroidal thickness (SFCT) was measured manually by two independent examiners (NV and SF), blinded to patients’ characteristics, using the caliper function tool of the image analysis software. We used for the statistical analysis the mean of the two measurements.

**Figure 2:**
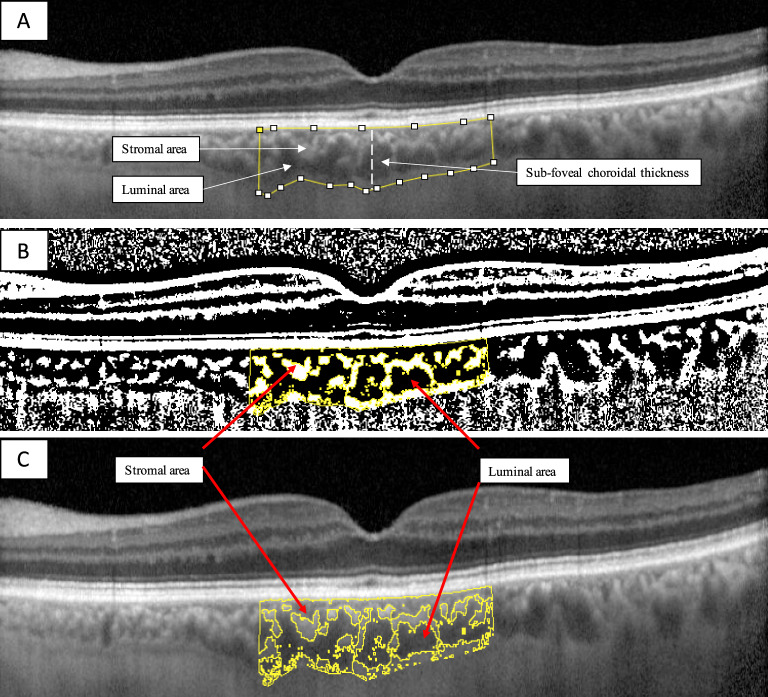
Choroidal vascular index calculation with binarization of enhanced depth imaging (EDI) spectral domain-optical coherence tomography (SD-OCT) images. (**A**) A region of interest of 3000 µm wide centered on the fovea was selected in the software ImageJ. The sub-foveal choroidal thickness (SFCT) was calculated. (**B**) The image was binarized using Niblack’s auto-local threshold. (**C**) The color threshold tool was used to select the dark pixels, representing the luminal choroidal area (LCA). The stromal choroidal area was calculated by subtracting LCA from the total choroidal area (TCA). The CVI was calculated by dividing LCA by TCA.

To assess the choroidal vascular index (CVI) OCT images were opened in the free software ImageJ 1.51 s (National Institutes of Health, Bethesda, MD). To assess a larger area of the choroid, we selected a region of interest of 3000 µm wide centered on the fovea. The upper boundary of the region of interest was traced along the choroidal–retinal pigment epithelium junction and the lower boundary along the choroidal–scleral junction to identify the total choroidal area (TCA). After conversion to an 8-bit image, Niblack’s auto-local threshold was applied to binarize the images and demarcate the luminal choroidal area (LCA) and the stromal choroidal area (SCA). The images were converted back to red, green, and blue images, and the color threshold tool was used to select the dark pixels, representing the LCA. The TCA and LCA were measured. The SCA was calculated by subtracting LCA from TCA. The CVI, defined as the LCA divided by the TCA, was then calculated. Two investigators performed the calculation of the choroidal parameters separately, blinded to patients’ characteristics (NV and SF). The mean value for each calculated parameter was used for the statistical analysis. See Fig. 2.

### Statistical analysis

For the statistical analysis, the eye with the best OCT image resolution was selected for the analysis. Normality was tested with the Shapiro–Wilk test. As variables were normally distributed, they were reported as mean and standard deviation (SD), and parametric tests were used in the statistical analysis.

The Chi-square test was used to measure the association between two categorical variables.

The inter-rater reliability for the image binarization was measured by the absolute agreement model of the intra-class correlation coefficient (ICC). ICC values less than 0.5, 0.5 and 0.75, 0.75 and 0.9, and greater than 0.90 were considered indicative of poor, moderate, good, and excellent reliability respectively, as previously described^14^. A Paired T-test was used to compare choroidal parameters, BCVA, and intraocular pressure (IOP) in iNPH before and after VP shunt surgery. Pearson coefficient was used to correlate IOP and choroidal thickness.

T-Test was used to compare demographic data, SFCT, TCA, LCA, SCA, and CVI between iNPH and healthy patients.

*p* values < 0.05 were considered statistically significant. Statistical analysis was performed using IBM Statistical Package for Social Sciences version 26.

### Ethics approval and consent to participate

All participants gave written informed consent. The study was approved by the local ethics committee of the local health service of Bologna, Italy (Cod CE: 809/2021). The patients did not receive any financial compensation for their participation. The patients were advised that the data collected were used for scientific purposes and they gave their consent for publication.

## Results

### Demographic characteristics

Of the 20 iNPH patients recruited a total of 18 eyes of 18 iNPH patients were considered for the analysis. One patient was excluded from the study because of laser-treated diabetic retinopathy in both eyes, and one because of the poor quality of OCT images, secondary to bilateral advanced cataracts. iNPH patients demographics were comparable to the control group using the T-test (*p* > 0.05). Two patients had a diagnosis of Open-Angle Glaucoma, representing the 11.1% of the total iNPH patients. Demographic data of iNPH patients and the control group are listed in Table 1. See Table 1.

**Table 1:** Demographic data of iNPH patients and the control group are shown.

	iNPH patients (n 18)	Control group (n 18)	*p* value
Age (mean, SD)	77.16 (± 5.99)	76.26 (± 5.20)	0.564
Sex (woman, %)	5 (27.8%)	8 (44.4%)	0.489
Cataract surgery n (%)	5 (27.8%)	5 (27.8%)	1
IOP (mean, SD)	14.90 (± 4.12)	15.73 (± 4.69)	0.588
BCVA (mean, range)	0.1 (0.4–0)	0.1 (0.4–0)	0.614

*SD *standard deviation.

There were no correlations between IOP and choroidal thickness (ρ = 0.114, *p* = 0.520) using the Pearson coefficient analysis.

### Primary objective

**Figure 3:**
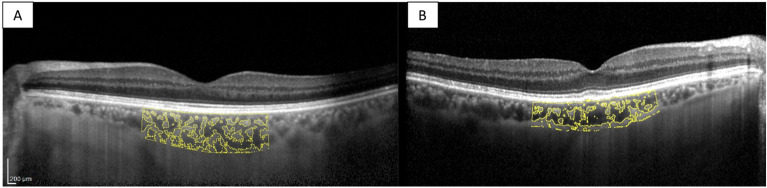
Choroidal morphology in nonshunted iNPH patients and healthy control. The sub-foveal region of 3000 μm is separated into the luminal choroidal area (LCA), and the stromal choroidal area (SCA). (**A**) Nonshunted iNPH eye. (**B**) Healthy control eye.

**Figure 4:**
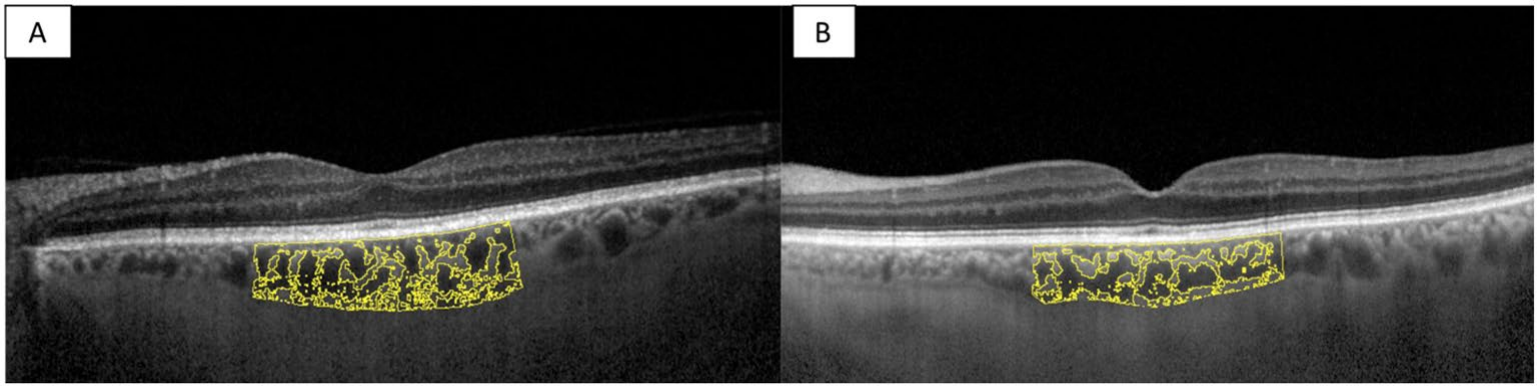
Choroidal morphology in shunted iNPH patients and healthy control. The sub-foveal region of 3000 μm is separated into the luminal choroidal area (LCA), and the stromal choroidal area (SCA). (**A**) Shunted iNPH eye. (**B**) Healthy control eye.

**Figure 5:**
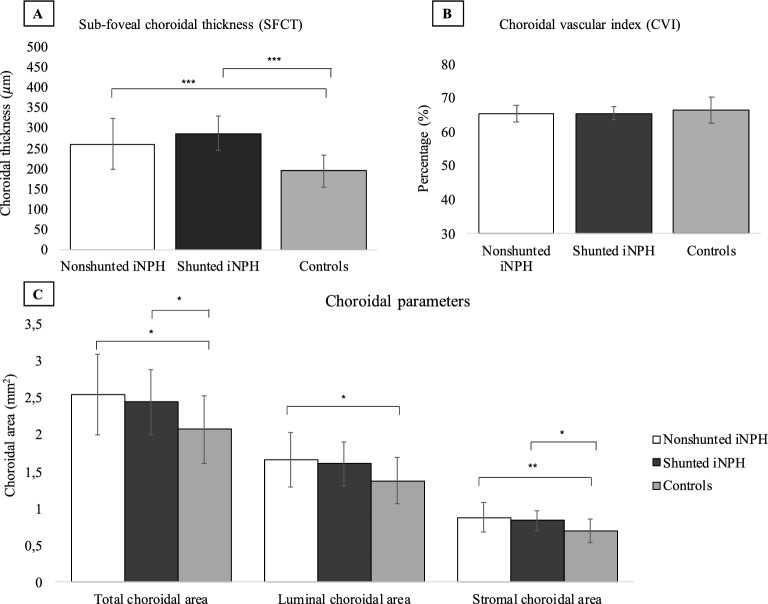
Choroidal parameters in nonshunted iNPH, shunted iNPH, and controls. (**A**) Sub-foveal choroidal thickness (SFCT) was 194 μm ± 40.58 in controls (n = 18), whereas it was 260 μm ± 63 μm in nonshunted iNPH (n = 18) *p* < 0.001), and 287 μm ± 42.80 μm in shunted iNPH patients (n = 10) (*p* < 0.001). (**B**) Choroidal vascular index (CVI) was not significantly different between nonshunted iNPH, shunted iNPH, and controls (65.45% ± 0.02, 65.56% ± 2.05, and 66.39% ± 3.92 respectively, *p* value > 0.05). (**C**) Nonshunted iNPH patients showed a statistically significant increase in the total choroidal area (TCA), luminal choroidal area (LCA), and stromal choroidal area (SCA) compared to controls (2.55 ± 0.55 vs. 2.08 ± 0.46 mm^2^, *p* = 0.011; 1.66 ± 0.37 vs. 1.38 ± 0.31 mm^2^, *p* = 0.020; 0.88 ± 0.20 vs. 0.70 ± 0.16 mm^2^, *p* = 0.006). Shunted iNPH patients showed statistically significant increase in the total choroidal area (TCA), and stromal choroidal area (SCA) compared to controls (2.45 ± 0.44 vs. 2.08 ± 0.46 mm^2^, *p* = 0.049; 0.84 ± 0.14 vs. 0.70 ± 0.16 mm^2^, *p* = 0.027). The LCA was increased in shunted iNPH patients compared to controls (1.61 ± 0.30 vs. 1.36 ± 0.31), even though the result was not statistically significant (*p* = 0.081).

iNPH patients before surgery compared to the control group showed a statistically significant increase in SFCT (260 ± 63 μm vs. 194 ± 40 μm, *p* < 0.001), TCA (2.55 ± 0.55 mm^2^ vs. 2.08 ± 0.46 mm^2^, *p* = 0.011), LCA (1.66 ± 0.30 vs. 1.38 ± 0.31 mm^2^, *p* = 0.020), and SCA (0.88 ± 0.20 vs. 0.70 ± 0.16 mm^2^) using the T-test analysis. Moreover, iNPH patients after VP shunt surgery compared to healthy individuals showed a statistically significant increase of SFCT (287 ± 42.8 vs. 194 ± 40.58 μm, *p* < 0.001), TCA (2.45 ± 0.44 vs. 2.08 ± 0.45 mm^2^, *p* = 0.049), and SCA (0.85 ± 0.14 vs. 0.70 ± 0.16 mm^2^, *p* = 0.027) using the T-test analysis. The LCA was increased in shunted iNPH patients compared to healthy controls using the T-test analysis (1.61 ± 0.30 vs. 1.38 ± 0.31 mm^2^), even though the result was not statistically significant (*p* = 0.081). The CVI was not statistically significant comparing nonshunted iNPH patients and shunted iNPH patients to controls using the T-test analysis (65.45% ± 0.02 vs. 66.39% ± 3.92, *p* = 0.492. 65.56% ± 2.05 vs. 66.39% ± 3.92, *p* = 0.472 respectively). Representative swept-source OCT images of the eyes of a nonshunted iNPH patient and a healthy control, and a shunted iNPH patients and a different healthy control are shown in Figs. 3 and 4. Summary data are shown in Fig. 5.

### Secondary objective

**Figure 6:**
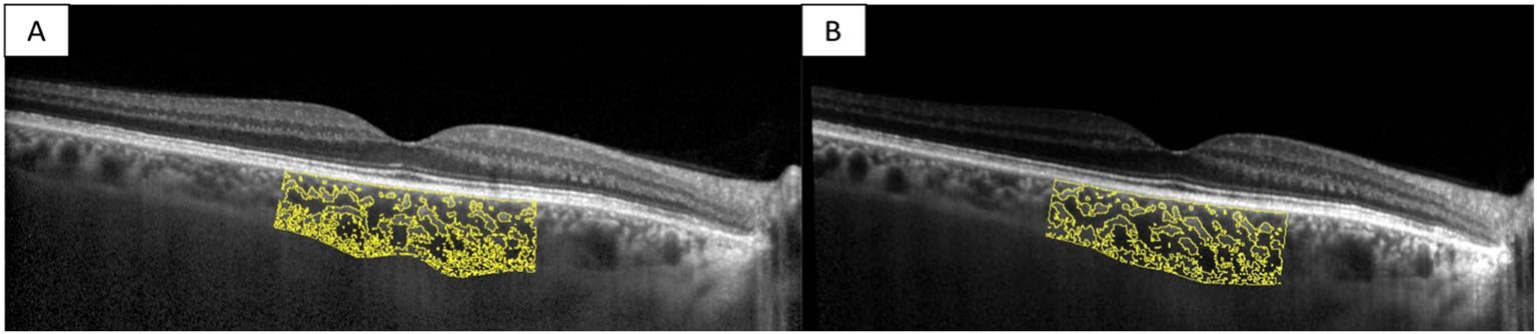
Choroidal morphology in iNPH patients before and after shunt surgery. The sub-foveal region of 3000 μm is separated into the luminal choroidal area (LCA), and the stromal choroidal area (SCA). (**A**) iNPH patient before shunt surgery. (**B**) iNPH patient after shunt surgery.

**Figure 7:**
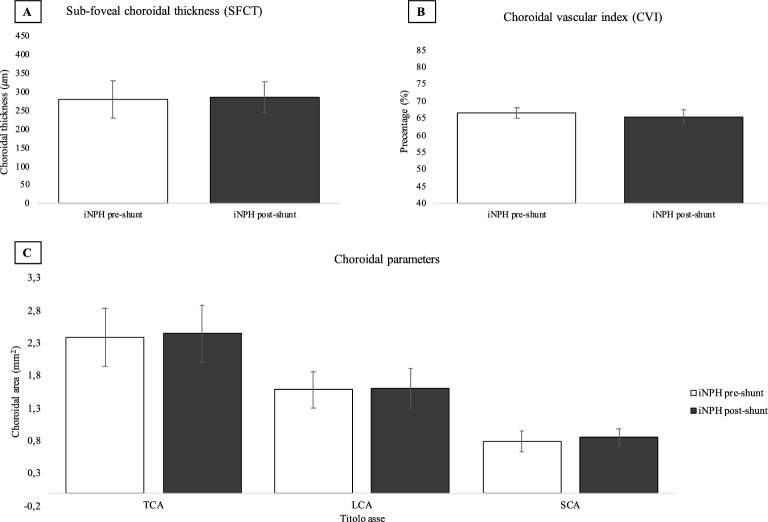
Choroidal parameters in the eyes of iNPH patients before and after shunt surgery. (**A**) Sub-foveal choroidal thickness (SFCT) was not significantly different before surgery (n = 10), and after surgery (n = 10) (281 ± 51.53 μm vs. 287 ± 42.80 μm, p = 0.223). (b) Choroidal vascular index (CVI) was not significantly different before surgery and after surgery (66.7% ± 1.51 vs. 65.5% ± 2.05, *p* = 0.107). (**C**) Total choroidal area (TCA), luminal choroidal area (LCA), and stromal choroidal area (SCA) were not significantly different before and after surgery (2.39 ± 0.44 vs. 2.45 ± 0.44 mm^2^, *p* = 0.676; 1.59 ± 0.28 vs. 1.61 ± 0.30 mm^2^, *p* = 0.885; 0.801 ± 0.16 vs. 0.845 ± 0.14 mm^2^, *p* = 0.245 respectively).

Of the 18 patients included, 10 patients underwent VP shunt surgery after a mean time from baseline ophthalmological evaluation of 48 days (SD ± 50.7). Then, iNPH patients were re-evaluated after a mean follow-up time of 56 days (SD ± 25.4). Visual acuity and IOP were not statistically different before and after shunt surgery using the paired T-test analysis (0.1 ± 0.07 vs. 0.1 ± 0.06, *p* = 0.443; 17.6 ± 3.65 mmHg vs. 16.9 ± 2.89 mmHg, *p* = 0.225 respectively). The choroidal structure was assessed after VP shunt surgery. iNPH patients before and after shunt surgery showed no statistical differences in CVI (66.7% ± 1.51 vs. 65.5% ± 2.05, *p* = 0.107), SFCT (281 ± 51.53 μm vs. 287 ± 42,80 μm, *p* = 0.223), TCA (2.39 ± 0.44 vs. 2.45 ± 0.44 mm^2^, *p* = 0.676), LCA (1.59 ± 0.28 vs. 1.61 ± 0.30 mm^2^, *p* = 0.885) and SCA (0.801 ± 0.16 vs. 0.845 ± 0.14 mm^2^, *p* = 0.245) using the paired T test analysis. Representative swept-source OCT images of a patient pre- and post-shunt are shown. See Fig. 6. Summary data are shown in Fig. 7.

### Inter-rater reliability analysis

The ICC analyses showed good to excellent inter-rater reliability between measurements for all choroidal parameters assessed. The results of the ICC analyses are shown. See Supplementary File 1.

## Discussion

The main finding of our study was that the choroid was statistically thicker in iNPH patients before and after VP shunt compared to age-matched healthy individuals. Moreover, we observed a statistically significant increase in the stromal area in iNPH patients before and after shunt surgery compared to the healthy controls. The luminal area was statistically greater in nonshunted iNPH patients compared to controls, and greater in shunted iNPH patients compared to controls, even though the result was not statistically significant. Overall, the CVI was similar in the two groups. In addition, we did not observe a significant statistical difference in the choroidal microstructure in the eyes of iNPH patients before and after a mean of 3 months from VP shunt surgery.

Anatomically, the choroid is made of five different layers: Bruch's membrane, the choriocapillaris, the two vascular layers (Haller's and Sattler's), and the suprachoroidal lamina. The main function of the choroid is to provide oxygen and nourishment to the outer layers of the retina. However, the choroid does not have autoregulation, even though a neural control is present, as the arterioles in the Sattler’s layer present vascular smooth muscle cells that are innervated by both divisions of the autonomic nervous system, that are implicated in the regulation of the blood flow in the choroidal circulation^15^. Previous studies found an increased superficial venous pressure and reduced venous drainage secondary to ventricular dilation in patients with iNPH, in a context of impaired CSF dynamic^16–18^. As the choroidal vasculature drains into the vortex veins and then into the cavernous sinus, a reduced venous outflow from the choroid could determine an accumulation of blood in the choroid and a subsequent increased choroidal thickness detected by OCT scan. This mechanism could explain the increased choroidal thickness observed in our study in iNPH patients compared to controls. However, we found no significant modifications in the choroid after a mean of 3 months from surgery in iNPH patients, suggesting a limited effect of VP shunting in the modification of the choroidal vasculature at 3 months from surgery. However, we cannot exclude that a longer follow-up could show different results.

In a previous retrospective study, Afonso et al. using SD-OCT reported lower values of SFCT and peripapillary choroidal thickness in 6 iNPH patients before shunt surgery compared to 13 healthy individuals. Moreover, their study reported higher values of SFCT in 6 iNPH patients after shunt surgery compared to 6 different non-shunted iNPH patients, suggesting a correlation between SFCT and cortical venous compliance^8^. In contrast with their findings, we found higher values of SFCT in 18 eyes of 18 non-shunted iNPH patients compared to healthy controls (260 μm ± 63 μm in nonshunted iNPH patients and 194 μm ± 40 μm in the control group, *p* < 0.001). Furthermore, we found higher values of SFCT in 10 eyes of 10 shunted iNPH patients compared to the healthy controls (287 μm ± 42.80 μm in shunted iNPH patients and 194 μm ± 40 μm in the control group, *p* < 0.001). Moreover, we did not observe significant statistical variations in SFCT in 10 iNPH patients prospectively assessed before and after a mean of 3 months from VP shunt surgery (281 ± 51.53 μm vs. 287 ± 42.80 μm respectively, *p* = 0.223). However, in the study by Afonso et al., they compared separate patients with iNPH who had or not had undergone shunt surgery, and the analysis was not performed in the same patients before and after shunt surgery, whereas in the present study the same patients were prospectively recruited before and after VP shunt surgery.

Interestingly, Afonso et al. reported lower values of peripapillary choroidal thickness in nonhunted iNPH patients compared to controls. As peripapillary choroidal thinning has been associated with an increased risk of normal tension glaucoma (NTG), and previous studies reported an increased risk of NTG in iNPH patients, future studies should be conducted to assess if there is a correlation between peripapillary choroidal thinning and the development of glaucomatous damages in iNPH patients^19–22^.

Another result of the present study was that the stroma in the choroid was increased in shunted and non-shunted iNPH patients compared to healthy controls. The choroidal stroma is composed of neural tissues, melanocytes, fibroblasts, macrophages, dendritic cells, and other extracellular components^23^. As previously described, an increased stromal area is linked to stromal inflammation in the choroid^24–27^. As the accumulation of neurotoxins and cytokines in the CSF, blood–brain barrier breakdown, and microvascular dysregulation are some of the hypotheses implicated in the pathogenesis of iNPH, we hypothesize that the increased stromal area could be an optical coherence tomography sub-clinical finding of inflammation occurring in the choroid, secondary to the accumulation of cytokines in the choroidal vasculature^6,7^. However, it is worth mentioning that none of these patients presented clinical signs of choroiditis or long-term sequelae of choroidal inflammation. Therefore, further studies are needed to confirm and better understand our findings, assessing the choroidal microstructure in other neuroinflammatory diseases.

Moreover, we found that the LCA was greater in both nonshunted and shunted iNPH patients compared to controls, even though this difference was not statistically significant in shunted patients compared to controls.

Another result of the present study is that we found no variations in the CVI between iNPH and healthy controls, indicating that the ratio between the total choroidal area and the vascular area was preserved.

The characterization of the choroidal structure in neurodegenerative diseases had been previously conducted with conflicting results. A previous study by Satue et al. reported increased choroidal thickness in eyes of patients with Parkinson’s disease (PD) compared to controls^28^. However, other studies reported a thinning of the choroid in PD patients compared to controls, suggesting the role of choroidal hypoperfusion as causing choroidal thinning in PD^29^.

A recent study by Robbins et al. reported an increased TCA, LCA, and reduced CVI in PD patients compared to controls, suggesting dysregulation of neurotransmitters, which control normal choroidal perfusion, as the primary cause for choroidal alterations^30^.

Moreover, previous studies reported a thinning of the choroid in eyes of patients with Alzheimer’s Disease (AD), compared to controls^31,32^. However, a recent histopathological study by Asanad et al., reported an increased choroidal thickness in the macular region in patients with severe AD, with an increased stromal area compared to controls, suggesting a compensatory vascular proliferation in the choroid in response to metabolic dysfunction in AD^33^. To the best of our knowledge, this is the first study to assess the choroidal microvasculature structure using the CVI measurement in patients with iNPH. The results of our findings support the hypothesis of choroidal involvement in patients with iNPH. Further studies are needed to confirm our findings, and to understand the role of OCT scan as a non-invasive tool in the assessment of patients with iNPH.

### Strengths and limitations

This is a prospective study with iNPH patients before and after VP shunt surgery and collected good-quality OCT images both from iNPH patients and age and sex-matched healthy control individuals.

The main disadvantage of using CVI consists of post-processing the images into external software for imaging elaboration. Hitherto, it has not been validated an automated algorithm which can calculate CVI from OCT images. The strength of the present study was that the choroidal parameters were measured by two independent examiners, blinded to patients’ characteristics, and the mean of the two measurements was used for the statistical analysis. Moreover, an inter-reliability analysis was undertaken and showed good to excellent results for each parameter considered.

A large number of studies have confirmed that age, diurnal variations, and axial length are the primary factors influencing choroidal thickness. In our study, we included patients and healthy individuals of similar age and AL < 26 mm, to avoid the presence of confounding factors, and each measurement was performed between 11.00 and 14.00 to avoid diurnal variations. We decided to choose a region of interest of 3000 μm to include a larger area of the choroid and not limit the analysis to the sub-foveal region.

The main limitation of our study is the small cohort of patients included. Second, although the interobserver agreement was good to excellent, we manually measured the SFCT and the TCA, which can be affected by the operator.

Also, another important limitation of our study is the short follow-up from VP shunt surgery.

Our future objective is to increase the number of iNPH patients with a longer follow-up to confirm our findings and eventually clarify the role of shunt surgery in the alterations of the choroidal structure.

## Conclusions

SFCT, TCA, and SCA showed higher values in iNPH patients compared to the control group both before and after shunt surgery. However, we did not observe a significant statistical difference in the choroidal microstructure in the eyes of iNPH patients before and after a mean of 3 months from VP shunt surgery. To the best of our knowledge, this is the first study to assess the choroidal microvasculature structure in patients with iNPH. The results of our findings support the hypothesis of choroidal involvement in patients with iNPH. Moreover, our results highlight the role of OCT as a non-invasive tool in the assessment of patients with iNPH.

### Supplementary Information

Supplementary Information.

## Data Availability

The datasets used and analyzed during the current study are available from the corresponding author upon reasonable request.

## Abbreviations

iNPH: Idiopathic normal pressure hydrocephalus
OCT: Optical Coherence Tomography
SD-OCT: Spectral-domain optical coherence tomography
EDI: Enhanced depth imaging
VP: Ventriculo-peritoneal
SFCT: Sub-foveal choroidal thickness
TCA: Total choroidal area
LCA: Luminal choroidal area
SCA: Stromal choroidal area
CSF: Cerebrospinal fluid
CVI: Choroidal vascular index
AL: Axial length
BCVA: Best-corrected visual acuity
LogMar: Logarithm of the minimum angle of resolution
SD: Standard deviation
ICC: Intra-class correlation coefficient
IOP: Intraocular pressure
PD: Parkinson’s disease
AD: Alzheimer’s Disease
NTG: Normal-tension glaucoma
