# Supplementary material for: Choroidal morphology and microvascular structure in eyes of patients with idiopathic normal pressure hydrocephalus before and after ventriculo-peritoneal shunt surgery
Source: Sci Rep. 2023 Sep 29;13:16379. doi: 10.1038/s41598-023-43518-8 (PMC10541413; doi:10.1038/s41598-023-43518-8)
Supplement: Supplementary file 1 — Supplementary Information. [file 41598_2023_43518_MOESM1_ESM.docx]

**Table 5**. Results of inter-reliability analysis for each choroidal parameter considered.

|  | **ICC** | **95% confidence interval (max-min)** | |
| --- | --- | --- | --- |
| SFCT 𝜇m, mean (SD) | 0,984 | 0,992 | 0,968 |
| TCA, mean (SD) | 0,894 | 0,942 | 0,809 |
| LCA, mean (SD) | 0,905 | 0,947 | 0,828 |
| SCA, mean (SD) | 0,872 | 0,929 | 0,768 |
| CVI, mean (SD) | 0,885 | 0,936 | 0,792 |
